# Metal ion-enhanced photosynthetic bacteria for bioplastic production from kitchen waste digestate: performance and mechanism

**DOI:** 10.3389/fmicb.2025.1645573

**Published:** 2025-08-12

**Authors:** Wei Zhao, Huize Liu, Jingyi Li, Shiqi Liu, Xue Tao, Li Sun, Longyi Lv, Jinsong Liang, Guangming Zhang

**Affiliations:** ^1^School of Heilongjiang River and Lake Management, Heilongjiang University, Harbin, China; ^2^School of Energy & Environmental Engineering, Hebei University of Technology, Tianjin, China; ^3^Department of Resources and Environment, Moutai Institute, Renhuai, Guizhou, China

**Keywords:** kitchen waste digestate, lactate, polyhydroxybutyrate, PhaC, metabolic characteristics

## Abstract

**Background:**

Polyhydroxybutyrate (PHB) production from food waste by photosynthetic bacteria (PSB) face the bottleneck of low production efficiency. Metal ions have the potential to enhance the PHB production by PSB. Thus, for the first time, this study explored the effect of Fe^3+^ and Mn^2+^ on the enhancement of PHB production from kitchen waste digestate by PSB and their enhancement mechanism.

**Methods:**

FeCl_3_·6H_2_O and MnCl_2_·4H_2_O were the main sources of Fe^3+^ and Mn^2+^. Five Hundred milliliter sealed Schott bottles were used as the fermentation reactor. Kitchen waste digestate was diluted to soluble chemical oxygen demand (SCOD) of 2.5 g/L as substrate and inoculated with 20% (v/v) mixed PSB. Fe^3+^ concentrations in these reactors were 10, 20, 30, and 40 mg/L, respectively. Mn^2+^ concentrations in these reactors were 1, 2, 3, and 4 mg/L, respectively. The initial pH of these reactors was adjusted to 8.0 and was carried out at room temperature of 26–30°C. All reactors were placed in a light-proof experimental chamber with a light intensity of 4,000 lx.

**Results:**

The optimal concentrations of 10 mg/L Fe^3+^ and 2 mg/L Mn^2+^ promoted PSB biomass and PHB accumulation, while excessive concentrations of metal ions inhibited them. Concentrations of PSB biomass reached 2366.3 and 2109.2 mg/L, respectively under the 10 mg/L Fe^3+^ and 2 mg/L Mn^2+^ concentrations, and PHB content reached 46.0 and 43.8%, respectively. Removal rate of SCOD and ammonia nitrogen in the kitchen waste digestate exceeded 90 and 70% under the 10 mg/L Fe^3+^ and 2 mg/L Mn^2+^ concentrations. The concentration of intracellular Fe^3+^ and Mn^2+^ that PSB adapts to growth was approximately 5.5 and 0.5 mg/L, respectively. The 10 mg/L Fe^3+^ and 2 mg/L Mn^2+^ concentrations decreased the diversity, altered the composition, and enhanced functional metabolism of microbial communities.

**Conclusion:**

The concentration of 10 mg/L Fe^3+^ and 2 mg/L Mn^2+^ significantly enhanced PSB biomass and PHB accumulation (*p* < 0.05). Enhancement mechanism was to increase the relative abundance of key microorganisms, improve metabolic functions, and promote the expression of key functional genes. This study provides new ideas and insights for efficient production of PHB.

## 1 Introduction

Bioplastics are the preferred alternative to traditional petroleum-based plastics due to their easy degradability, renewability, and high safety (Patel et al., 2025). Polyhydroxybutyrate (PHB) are a class of biopolymers that are natural polyesters stored by microorganisms under stress conditions (Bhatia et al., 2024). PHB are widely recognized as a green and environmentally friendly polymer material, and can be applied in many fields such as bulk plastics, medical materials, biofuels, etc. and have been widely used in enhancement due to their diversity of types and properties (Liang et al., 2024b). However, the high cost of substrates hinders the PHB production with microbial methods (Zhang et al., 2025). Kitchen waste contains abundant organic matters, such as carbohydrates, proteins, etc. These organic matters can be converted into organic acids via anaerobic fermentation, which are high-quality substrates for PHB production (Amabile et al., 2024; Wang et al., 2024). Photosynthetic bacteria (PSB) are one of the potential microorganisms for PHB production, and can synthesize PHB under physiological stress conditions of nutrient deficiency or imbalance of redox (Zhang et al., 2024). Dan et al. (2023) reported that *Rhodopseudomonas palustris* could ferment kitchen waste digestate for PHB production, and PHB content reached 33% dry cell weight (DCW) during 35 d fermentation. Zhang et al. (2024) also reported that a mixed PSB microbial community achieved a PHB content of 41.6% DCW using kitchen waste digestate as a substrate. However, the production efficiency of PHB from kitchen waste is relatively low and needs further improvement.

Among the chemical, physical, and biological enhancements of PSB growth and product accumulation, trace elements as additives are simple, cheap, and effective (Jiang et al., 2023; Zhang et al., 2023). Essential metal ions (such as Fe^2+^/Fe^3+^, Zn^2+^, Cu^2+^, etc.) are key cofactors in various enzymes and electron transport chains, participating in key physiological processes such as microbial energy metabolism, DNA synthesis, and antioxidant defense (Verma et al., 2025; Wang et al., 2024). Some studies have been reported that metal ions can increase the utilization efficiency of organic matters and production of products in PSB (Montiel-Corona and Buitrón, 2025; Cao et al., 2020). Currently, metal ions such as Fe^2+^, Fe^3+^, Mg^2+^, etc. have been used to enhance PSB growth and product production. Zhang et al. (2024) found that Fe^2+^ and Mo^2+^ improved the productivity of H_2_ by PSB using wheat straw as a substrate, and H_2_ productivity was 48.1 mL/h/L. Montiel-Corona and Buitrón (2025) also reported that Fe^3+^ addition enhanced the simultaneous production of 5-ALA, coenzyme Q10, and pigments by PSB fermentation of residual wine lees. Thus, the addition of metal ions has the potential to enhance the PHB production by PSB. Currently, the effectiveness of metal ion enhanced PSB in producing PHB using kitchen waste digestate is not yet known. The enhancement mechanism of metal ions is also unclear.

Fe^3+^ and Mn^2+^ as essential metal ions play key cofactors and metabolic regulatory roles in bacteria, and have been widely used in enhancement of PSB growth and product accumulation (Liang et al., 2024b; Verma et al., 2025). Thus, Fe^3+^ and Mn^2+^ were selected to enhance PHB production by PSB in this study. The enhancement effect and mechanism of Fe^3+^ and Mn^2+^ on the PHB production by PSB using kitchen waste digestate were investigated for the first time. This study aims, specifically, (1) to explore the effects of different concentrations of Fe^3+^ and Mn^2+^ on the PSB biomass, PHB concentration, and PHB content, (2) to measure the soluble chemical oxygen demand (SCOD), lactate, and ammonia nitrogen concentrations in the kitchen waste digestate by PSB, (3) to clarify the diversity, community structure, and functional characteristics of PSB, and (4) to reveal the enhancement mechanism of Fe^3+^ and Mn^2+^ for PHB production by PSB. This study offers a new approach to enhance the PHB production by PSB using kitchen waste digestate and provides new insights of the enhancement mechanism.

## 2 Materials and methods

### 2.1 Material

To ensure the stability of kitchen waste, this study adopted manual configuration of kitchen waste, consisting of 40% rice, 10% noodles, 5% Mantou, 25% vegetables, 9% tofu, and 11% meat (Zhang et al., 2024). Kitchen waste was pulverized, then mixed with an appropriate amount of water, and finally placed in sealed bottles for natural anaerobic fermentation. Anaerobic fermentation was considered complete until the pH in the fermentation system dropped to about 4.0. The indices of kitchen waste before and after natural anaerobic fermentation were shown in Supplementary Table S1. Kitchen waste digestate was used as a substrate for subsequent PHB production. The basic properties of kitchen waste digestate were pH of 3.9, SCOD concentration of 56.4 g/L, ammonia nitrogen concentration of 0.21 g/L, and soluble protein concentration of 4.7 g/L. SCOD was mainly composed of soluble sugars, soluble proteins and small molecules of organic acids. Noted that lactate concentration, as the main organic acid, was 11.6 g/L. Thus, the type of acid-producing fermentation in natural anaerobic fermentation of kitchen waste was lactate type fermentation.

The inoculum used in this study was a mixed PSB community, purchased from Yancheng Bainuo Biotechnology Co., Ltd. *Rhodopseudomona* sp. was the dominant genus according to the 16S rRNA sequencing. PSB were activated in RCVBN medium, which contained sodium acetate (4.5 g/L), ammonium chloride (0.8 g/L), sodium bicarbonate (4 g/L), potassium dihydrogen phosphate (0.2 g/L), and yeast extract (0.5 g/L) (Liu et al., 2022). The inoculum was cultured at appropriate conditions and used for subsequent inoculation experiments when the PSB reached the logarithmic growth stage.

### 2.2 Experimental design

This study used 500 mL sealed Schott bottles as the photo-anaerobic fermentation reactors. Based on the result of preliminary experiment, 2.5 g/L of SCOD in kitchen waste digestate was the optimal concentration for PSB growth and PHB production. Thus, SCOD concentration of kitchen waste digestate was diluted to 2.5 g/L as substrate and inoculated with 20% (v/v) mixed PSB. Ferric chloride hexahydrate (FeCl_3_·6H_2_O) was added to the reactor to give Fe^3+^ concentrations of 10, 20, 30, and 40 mg/L, respectively. Similarly, manganese chloride tetrahydrate (MnCl_2_·4H_2_O) was added to the reactor to give Mn^2+^ concentrations of 1, 2, 3, and 4 mg/L, respectively. The initial pH of these reactors was adjusted to 8.0, magnetic stirrer rotation speed was 80 rpm, and photo-fermentation was carried out at room temperature of 26–30°C. The fermentation process was conducted without pH control in the reactor. To avoid the interference of natural light, all reactors were placed in a light-proof experimental chamber (0.6 m × 0.4 m × 0.4 m) with a light intensity of 4,000 lx (Supplementary Figure S1). For lighting, 12 W white LED strips with adjustable light intensity were implemented. Samples were taken from the reactor on days 1, 3, 5, 7, 9, 11, 13 and 15, respectively. Then, these samples were immediately centrifuged at 10,000 *g* for 10 min at 4°C after collection. The supernatant was used test for SCOD, ammonia nitrogen, and lactate concentrations. The precipitation after centrifugation were resuspended in an equal volume of deionized water and used to determine PSB biomass, PHB content, and intracellular ion concentration. Part of the precipitate was stored in −20°C refrigerator for subsequent microbial community analysis.

### 2.3 Analytical methods

The SCOD and ammonia nitrogen concentrations were determined according to standard methods (APHA, 2012). The concentration of lactate was measured using RQflex plus 10 kit (RQflex^®^plus 10, Germany) according to the manufacturer’s instructions. The intracellular Fe^3+^ and Mn^2+^ concentrations were measured by inductively coupled plasma-mass spectrometry (Agilent 8,800 ICP-MS/MS, Agilent Technologies, Tokyo, Japan). PSB biomass, PHB content, and PHB yield were determined based on the previous study (Zhang et al., 2025). Specifically, PSB biomass was tested using the optical density at 660 nm (OD660)-DCW method and the relationship between OD660 and DCW was biomass (mg/L, DCW) (Equation 1).


(1) PSBbiomass(mg/L)=434.8×OD660−10.3

For the detection of PHB content, the PSB biomass was freeze-dried for 48 h, then put in a closed reaction vessel with 2 mL of chloroform, 1.7 mL of methanol, and 0.3 mL of sulfuric acid. The container was placed in a 100°C water bath for esterification reaction, and the lower organic solvent was taken for PHB determination. PHB standard was purchased from Shanghai Macklin Biochemical Technology Co., Ltd. China. PHB content was determined using an external standard method for gas chromatography (GC-2018, Simazu (China) Ltd., China). The gas chromatogram of PHB standard and the representative PSB sample was shown in Supplementary Figure S2, confirming the PHB production. PHB yield was calculated by Equation 2.


(2) $$\mathrm{PHB}\;\text{yield}\;(g\;\mathrm{PHB}/g\;\mathrm{COD})=\frac{\text{Accumulated}\;\mathrm{PHB}\;\text{concentration}}{\text{Consumed}\;\mathrm{COD}\;\text{concentration}}$$

16S rRNA gene sequencing analysis was performed on the Shanghai Majorbio Technology Co., Ltd. and the specific sequencing process was described in detail in Supplementary Text S1 (Liang et al., 2024a,b; Zhang et al., 2025).

### 2.4 Statistical analysis

All experimental groups and indicators were tested three times in this study. Statistical significance was assessed by one-way ANOVA, with a threshold of *p* < 0.05 considered statistically significant.

## 3 Results and discussion

### 3.1 Effect of metal ions on PSB biomass and PHB accumulation

#### 3.1.1 Fe^3+^

**Figure 1:**
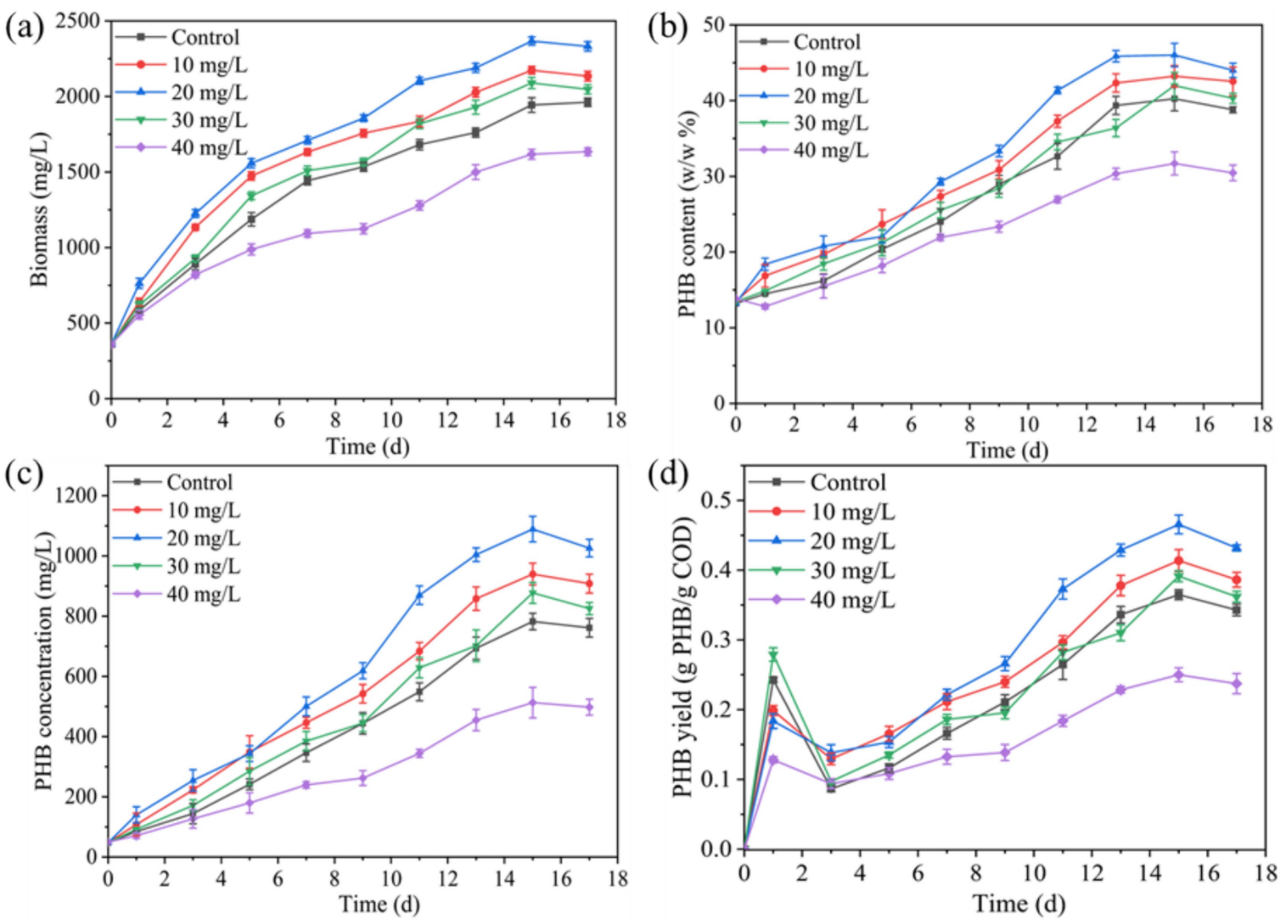
Effect of Fe^3+^ on changes in **(a)** PSB biomass, **(b)** PHB content, **(c)** PHB concentration, and **(d)** PHB yield.

Figure 1 demonstrates the enhancement effect of Fe^3+^ on PSB biomass and PHB accumulation in kitchen waste digestate. The concentration of 10–30 mg/L Fe^3+^ improved the PSB biomass, PHB content, PHB concentration, and PHB yield. Among them, the optimal Fe^3+^ concentration was 10 mg/L, and PSB biomass, PHB content, PHB concentration, and PHB yield reached 2366.3 mg/L, 46.0%, 1,089 mg/L, and 0.47 g-PHB/g-COD-removal, respectively. These indicators were significantly improved by 21.8, 14.4, 39.3 and 27.7%, respectively compared to the control (*p* < 0.05). In this study, the increase of PSB biomass (21.8%) and PHB content (14.4%) at 10 mg/L Fe^3+^ concentration were higher than the low-intensity ultrasound enhanced PSB biomass (17.9%) and PHB content (12.4%) reported by Zhang et al. (2025). Meanwhile, the PHB concentration in this study was significantly higher than that of photo-fermentation of wine lees by PSB reported by Montiel-Corona and Buitrón (2025). Nur et al. (2023) also reported that Fe^3+^ addition increased PHB production of *Synechocystis* sp. in treating palm oil mill effluent, but the PHB concentration was significantly lower than that of this study. Fe^3+^, as the core component of key electron carriers such as cytochromes and ferritin, can participate in the electron transport chain of the photosystem, and promote the efficiency of photosynthesis of PSB, further improving PSB growth and biomass production (Chaiyarat and Saejung, 2022; Yang and Wang, 2018). Meanwhile, Fe^3+^ can enhance key metabolic pathways and alleviate oxidative stress responses, further enhancing PHB production (Lu et al., 2019). However, high concentrations of Fe^3+^ (40 mg/L) significantly inhibited the PSB biomass and PHB accumulation. Chen et al. (2020) and Wu et al. (2012) also reported that more than 20 mg/L of nano zero-valent iron and Fe^2+^ significantly inhibited PSB biomass. This might be due to the toxicity of excess iron ion to microorganisms and further reduces enzyme activity (Wu et al., 2012).

#### 3.1.2 Mn^2+^

**Figure 2:**
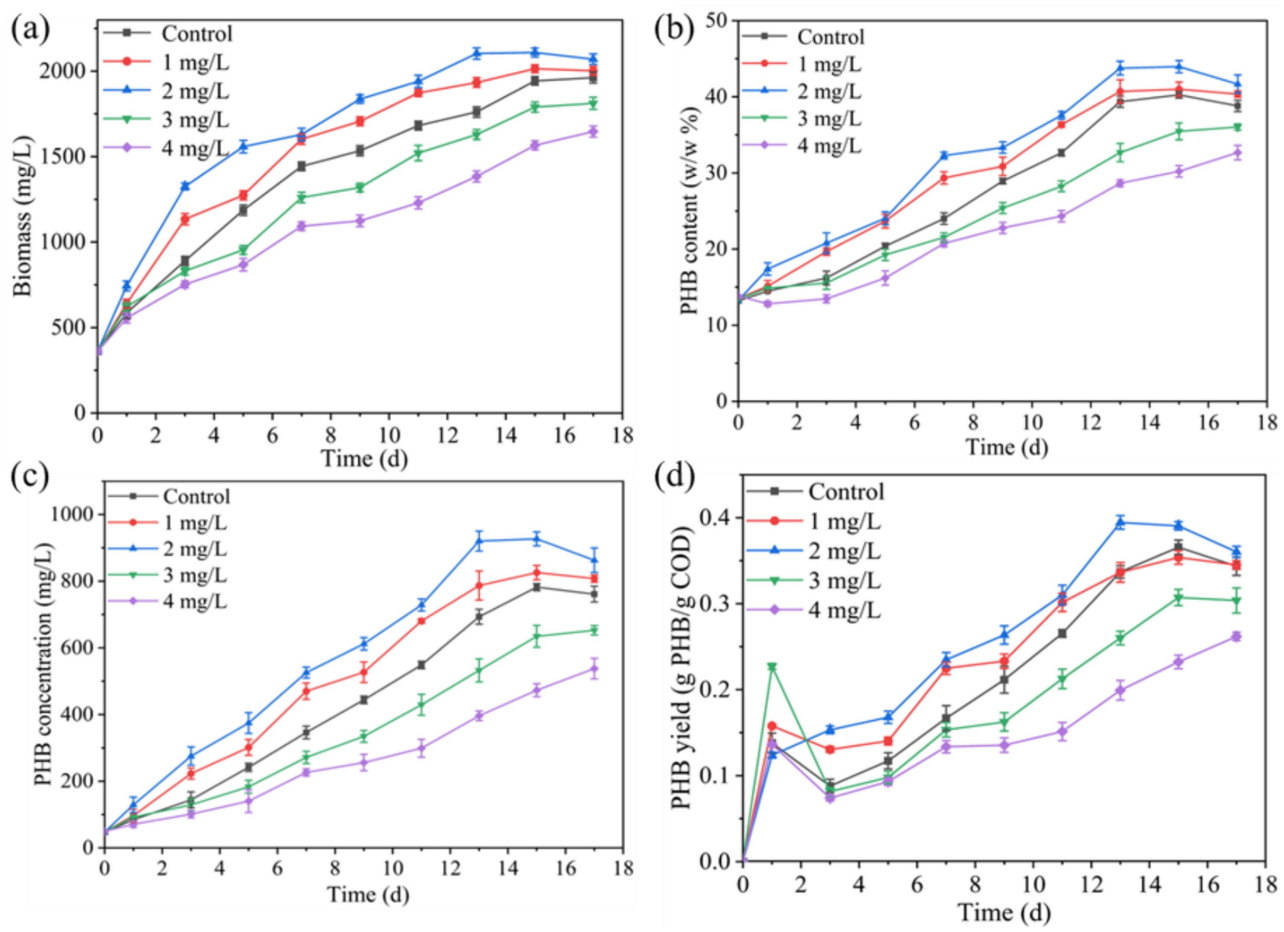
Effect of Mn^2+^ on changes in **(a)** PSB biomass, **(b)** PHB content, **(c)** PHB concentration, and **(d)** PHB yield.

Figure 2 demonstrates the enhancement effect of Mn^2+^ on PSB biomass and PHB accumulation in kitchen waste digestate. The concentration of 1 and 2 mg/L Mn^2+^ improved the PSB biomass, PHB content, PHB concentration, and PHB yield. Under the Mn^2+^ concentration of 2 mg/L, PSB biomass, PHB content, PHB concentration, and PHB yield reached 2109.2 mg/L, 43.8%, 920.2 mg/L, and 0.39 g PHB/g-COD-removal, respectively, which increased by 8.5, 4.4, 37.2, and 8.3% respectively, compared to the control. Mn^2+^ exhibited enhancement effect for PSB biomass similar to that of magnesium, iron, zinc, and cobalt ions (Liu et al., 2015; Liu et al., 2016). Thus, Fe^3+^ and Mn^2+^ significantly enhance the production of PHB from PSB. PSB biomass and PHB accumulation at 2 mg/L Mn^2+^ concentration were significantly lower than that of 10 mg/L Fe^3+^ concentration (*p* < 0.05). Strategies for PHB accumulation by Fe^3+^ and Mn^2+^-enhanced PSB was higher than that of *Calothrix scytonemicola* under specific inorganic salt regulation strategies (Kaewbai-ngam et al., 2016). Similar to Fe^3+^ group, Mn^2+^ can also activate the antioxidant defense system, enhance the activity of key metabolic enzymes, and improve photosynthetic efficiency (Lu et al., 2019), thereby increasing PSB biomass and PHB accumulation. Similar to the Fe^3+^ group, PHB yield also showed an initial increase followed by a decrease in the Mn^2+^ group during the early stages of fermentation. This might be due to the rapid consumption of SCOD in the early stages of fermentation for PSB biomass growth, and the slower consumption of SCOD in subsequent fermentation for PHB synthesis.

**Figure 3:**
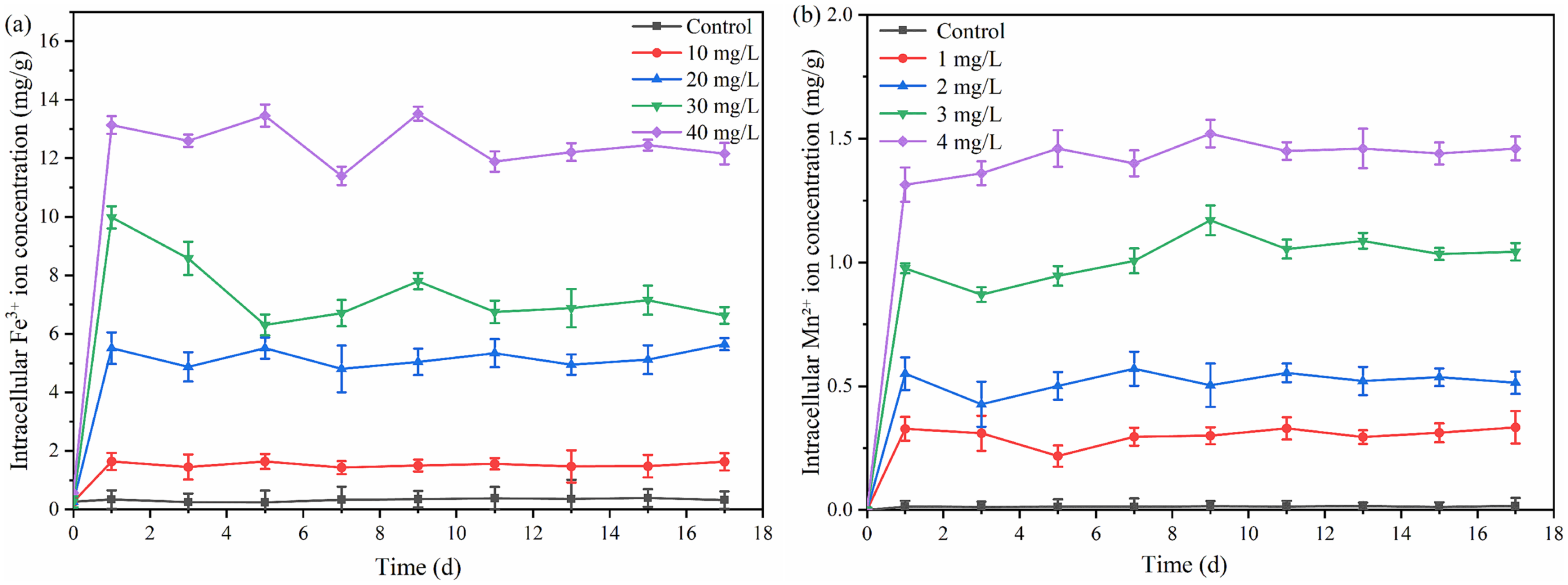
Changes of intracellular Fe^3+^ **(a)** and Mn^2+^ **(b)** in PSB under different ion concentrations.

Mn^2+^ concentration exceeding 3 mg/L significantly inhibited the PSB biomass and PHB accumulation (*p* < 0.05). This phenomenon was consistent with excessive Fe^3+^ (Figure 1). As shown in Figure 3, the concentration of intracellular Fe^3+^ and Mn^2+^ that PSB adapted growth was approximately 5.5 and 0.5 mg/L, respectively. Excessive intracellular metal ions can deteriorate microorganisms, affect the metabolic activity, and reduce the enzyme activity (Chen et al., 2020). Wu et al. (2012) also reported that the biomass of PSB was greatly affected when the intracellular Fe^2+^ concentration was above 16 mg/L. Therefore, the suitable ion concentrations are required to promote PSB growth and product accumulation.

### 3.2 Effect of metal ion-enhanced substrate utilization

**Figure 4:**
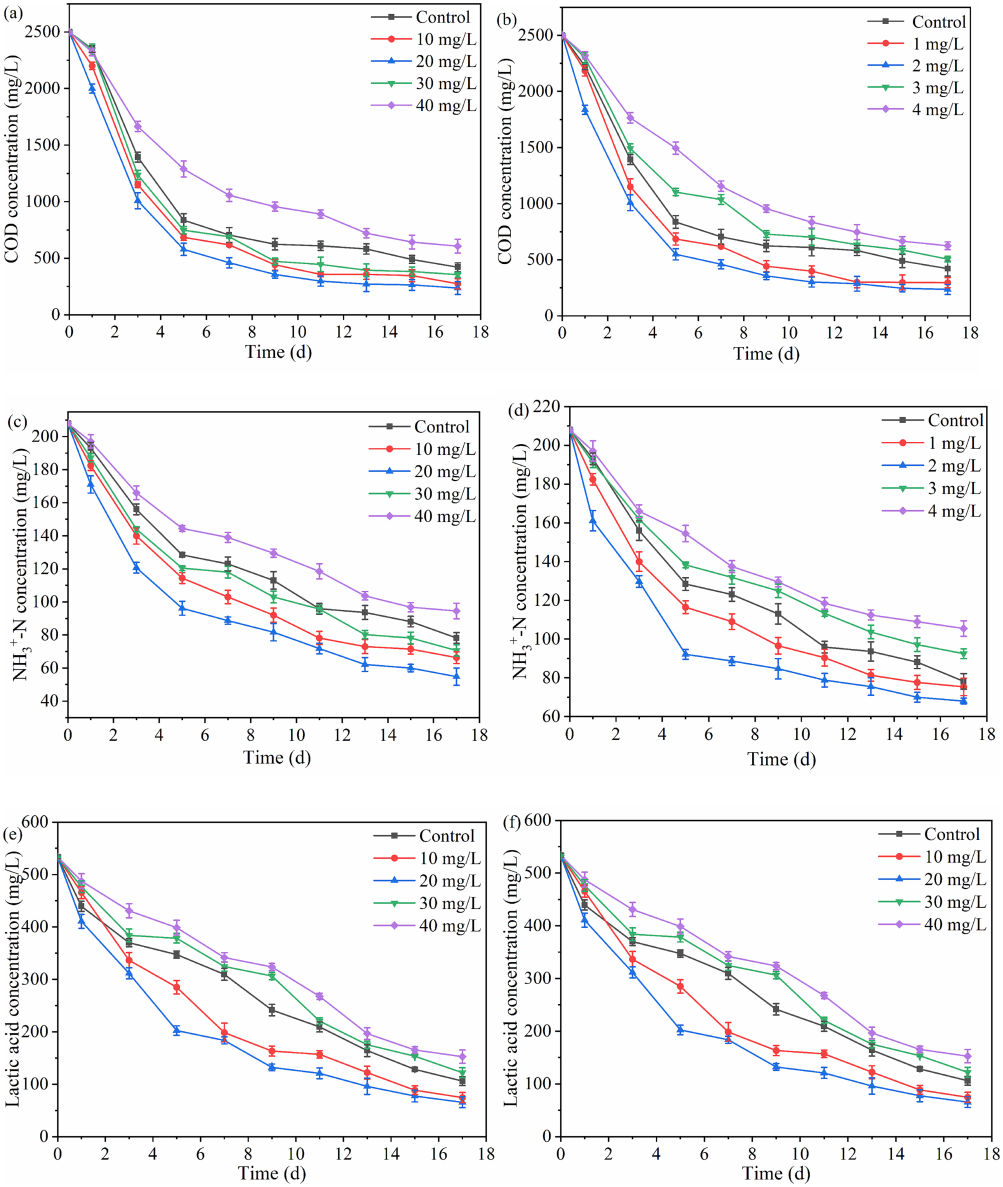
Effect of different Fe^3+^ and Mn^2+^ concentrations on the removal rate of organic matter. **(a,b)** SCOD concentration, **(c,d)** ammonia nitrogen concentration, and **(e,f)** lactate concentration.

Figure 4 shows the changes in substrate concentration for PHB production with PSB. As the fermentation time increased, the concentrations of SCOD, ammonia nitrogen, and lactate showed a significant decrease, indicating that the substrates were effectively utilized by PSB in all groups. Among them, SCOD, ammonia nitrogen, and lactate removal rate under 10–30 mg/L Fe^3+^ concentration were improved, corresponding to PSB biomass and PHB accumulation. Under the 10 mg/L Fe^3+^ concentration, the removal rate of SCOD, ammonia nitrogen, and lactate reached 90.5, 73.6, and 87.8%, respectively, which was increased by 7.3, 11.2, and 7.7% respectively, compared to the control (*p* < 0.05). Similarly, 1 and 2 mg/L Mn^2+^ concentration improved the removal rate of SCOD, ammonia nitrogen, and lactate. Under the 2 mg/L Mn^2+^ concentration, the removal rate of SCOD, ammonia nitrogen, and lactate reached 90.1, 67.3, and 87.6%, respectively, which was increased by 6.9, 4.9, and 7.6% respectively, compared to the control. These results were consistent with those reported by Chen et al. (2020). There was almost no difference in the removal rate of SCOD, ammonia nitrogen, and lactate under the optimal concentrations of Fe^3+^ and Mn^2+^. Meanwhile, excessive concentrations of Fe^3+^ and Mn^2+^ also inhibited the utilization of organic matter by PSB, further suppressing the PHB accumulation. This may be due to that the appropriate Fe^3+^ and Mn^2+^ concentrations can enhance the activity of key enzymes, improve photosynthetic efficiency, and further promote PSB growth (Chen et al., 2020), thereby increasing the utilization of substrates by PSB.

### 3.3 Microbiological analysis

#### 3.3.1 Diversity and richness

**Figure 5:**
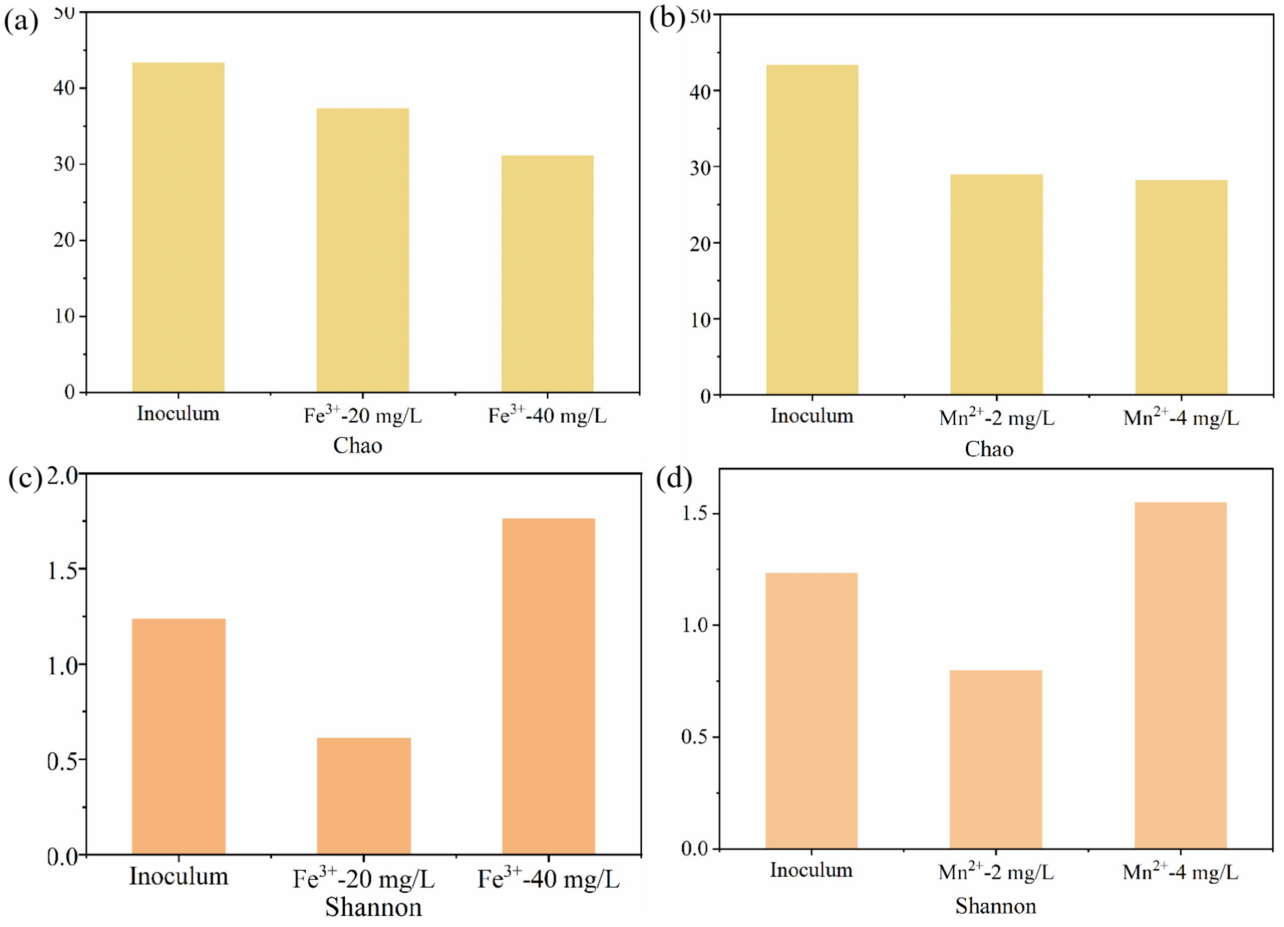
The effect of Fe^3+^ and Mn^2+^ on the microbial richness and diversity in photo-fermentation systems. **(a,c)** Chao index and **(b,d)** Shannon index.

Typically, the Chao and Shannon indices represent the richness and diversity of microbial communities (Song et al., 2025). The effects of Fe^3+^ and Mn^2+^ on the Chao and Shannon indices of microbial communities were consistent (Figure 5). Compared to the inoculum, appropriate Fe^3+^ and Mn^2+^ reduced the Chao and Shannon indices of microorganisms, while excessive Fe^3+^ and Mn^2+^ reduced the Chao and increased the Shannon indices of microorganisms. Meanwhile, the Chao index of 10 mg/L Fe^3+^ was higher than that of 2 mg/L Mn^2+^, while the Shannon index was lower than that of Mn^2+^ (Figure 5). These results indicate that appropriate metal ions can reduce microbial diversity, while excessively high metal ion concentrations increase microbial diversity. This may be because appropriate metal ions are more conducive to the growth of dominant microorganisms, while excessive metal ions breed the growth of miscellaneous bacteria, further increasing the diversity of microorganisms.

#### 3.3.2 Community composition

**Figure 6:**
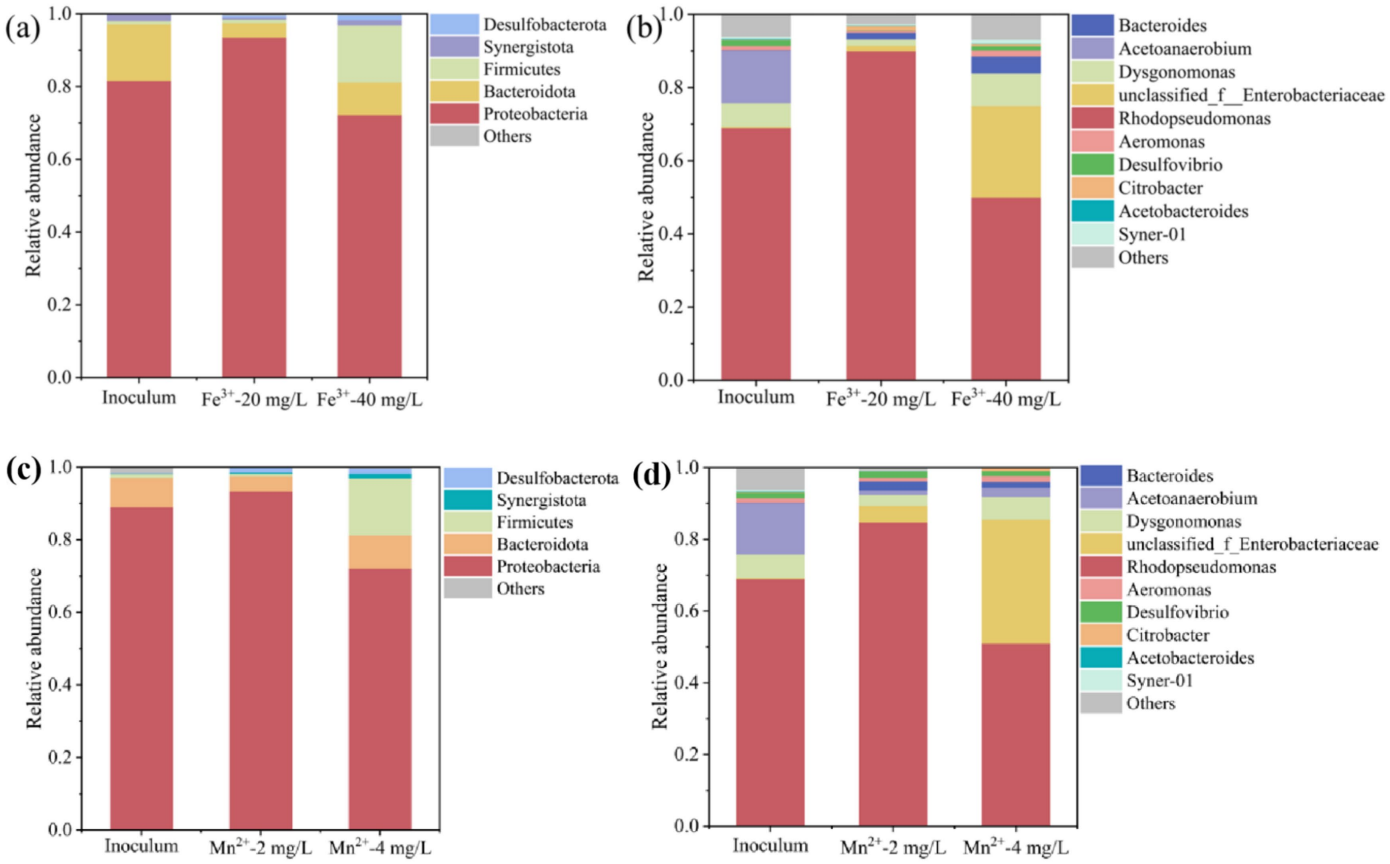
Effect of Fe^3+^ and Mn^2+^ on the microbial community composition of PSB at **(a,c)** phylum level and **(b,d)** genus level.

To investigate the effect of metal ions on microbial community composition, the community composition of PSB at the phylum and genus levels was analyzed. As shown in Figure 6, Proteobacteria, Firmicutes, and Bacteroidota were the main phyla in Fe^3+^ and Mn^2+^ photo-fermentation systems. Appropriate Fe^3+^ and Mn^2+^ increased the relative abundance of Proteobacteria, reaching 90.2 and 93.2%, respectively. Moreover, excessive Fe^3+^ and Mn^2+^ reduced the relative abundance of Proteobacteria, and greatly increased the relative abundance of Firmicutes.

As shown in Figure 6, *Rhodopseudomonas*, *Dysgonomonas*, and *Acetanaerobium* were the main genera in photofermentation systems. *Rhodopseudomonas* is one of the most typical PSB genera with strong environmental adaptability and shock resistance, and has been widely used to treat different types of organic wastewater to produce high value-added products, such as proteins, PHB, and coenzyme Q10, etc. (Carlozzi et al., 2019; Sun et al., 2023). In this study, the appropriate Fe^3+^ and Mn^2+^ significantly increased the relative abundance of *Rhodopseudomonas*, reaching 93.4 and 84.7%, and dominated the microbial community, corresponding to a higher PSB biomass and PHB accumulation under this condition. Zhang et al. (2025) reported that the higher relative abundance of *Rhodopseudomonas* corresponds to higher PHB production. Montiel-Corona and Buitrón, (2025) also found that suitable conditions promoted the relative abundance of *Rhodopseudomonas*, corresponding to PSB biomass and PHB accumulation. The excessive concentration of Fe^3+^ and Mn^2+^ significantly reduced the relative abundance of *Rhodopseudomonas*, which was also the main reason for inhibiting PSB biomass and PHB accumulation. In addition, the relative abundance of some genera significantly increased at the excessive concentration of Fe^3+^ and Mn^2+^, such as unclassified_f_Enterobacteriaceae, *Bacteroides*, etc. These genera may compete with *Rhodopseudomonas* for ecological niches, further inhibiting PSB growth and PHB accumulation.

Thus, an appropriate concentration of metal ions promoted the growth of *Rhodopseudomonas*, further increasing PSB biomass and PHB accumulation. While the excessive concentration of metal ions increased the growth of microorganisms other than PSB. These microorganisms competed with PSB and hindered the growth of *Rhodopseudomonas*, thereby inhibiting PSB biomass and PHB accumulation.

#### 3.3.3 Expression of functional genes

**Figure 7:**
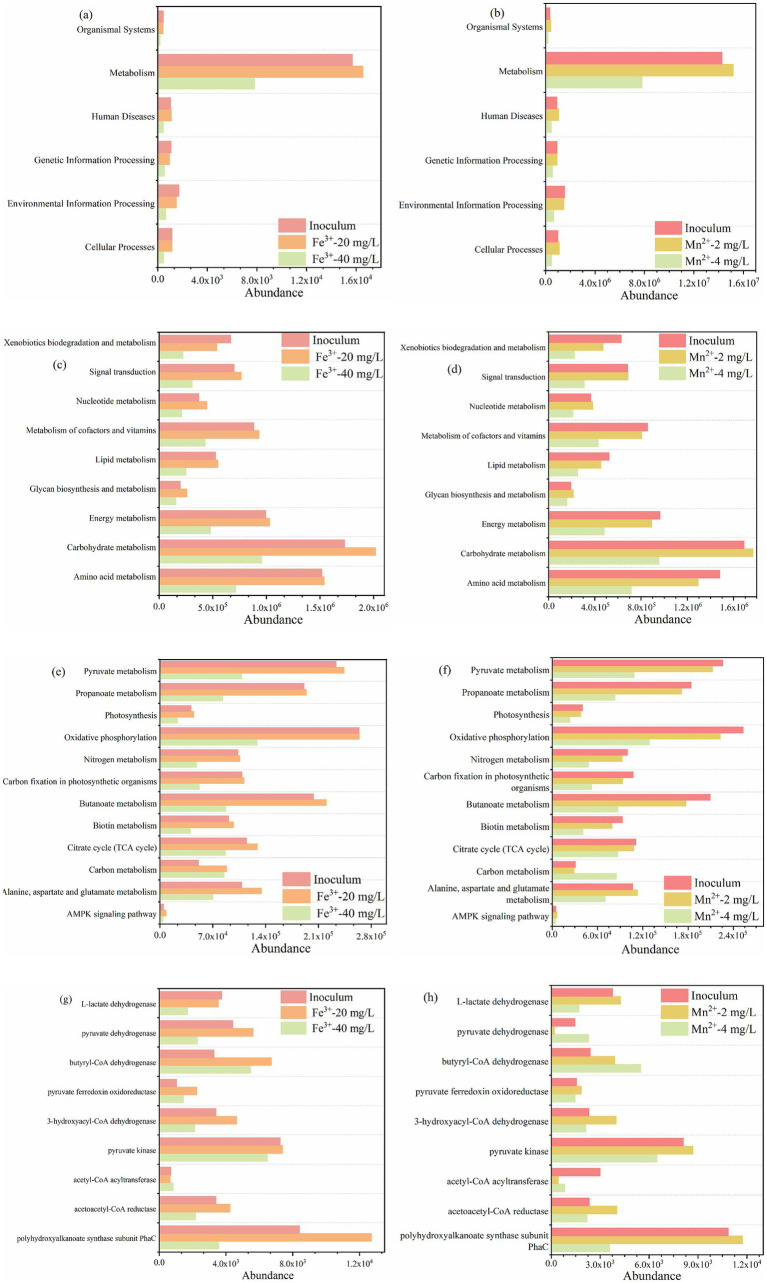
Changes in abundance of metabolic characteristics of PSB for PHB production with Fe^3+^ and Mn^2+^. **(a,b)** Level 1, **(c,d)** level 2, **(e,f)** level 3, and **(g,h)** functional genes.

To further analyze the enhancement mechanism of metal ions, the metabolic characteristics and functional gene expression of microorganisms were explored. From the first level, the appropriate concentration of metal ions significantly promoted metabolism, while excessive concentration of metal ions significantly inhibited almost all functional characteristics of microorganisms (Figures 7a,b). Carbohydrate metabolism and amino acid metabolism, which belong to metabolism, were the main metabolisms at secondary level metabolic pathway (Figures 7a,b), indicating that the appropriate concentration of metal ions significantly improved the C and N metabolism of PSB that utilize kitchen waste digestate to produce PHB. Meanwhile, metal ions increased the abundance of the TCA cycle, fatty acid metabolism, photosynthesis, pyruvate metabolism, etc. at third level metabolic pathway (Figures 7a,b). Therefore, metal ions promoted relevant metabolic functions at different levels, further promoting PHB accumulation.

**Figure 8:**
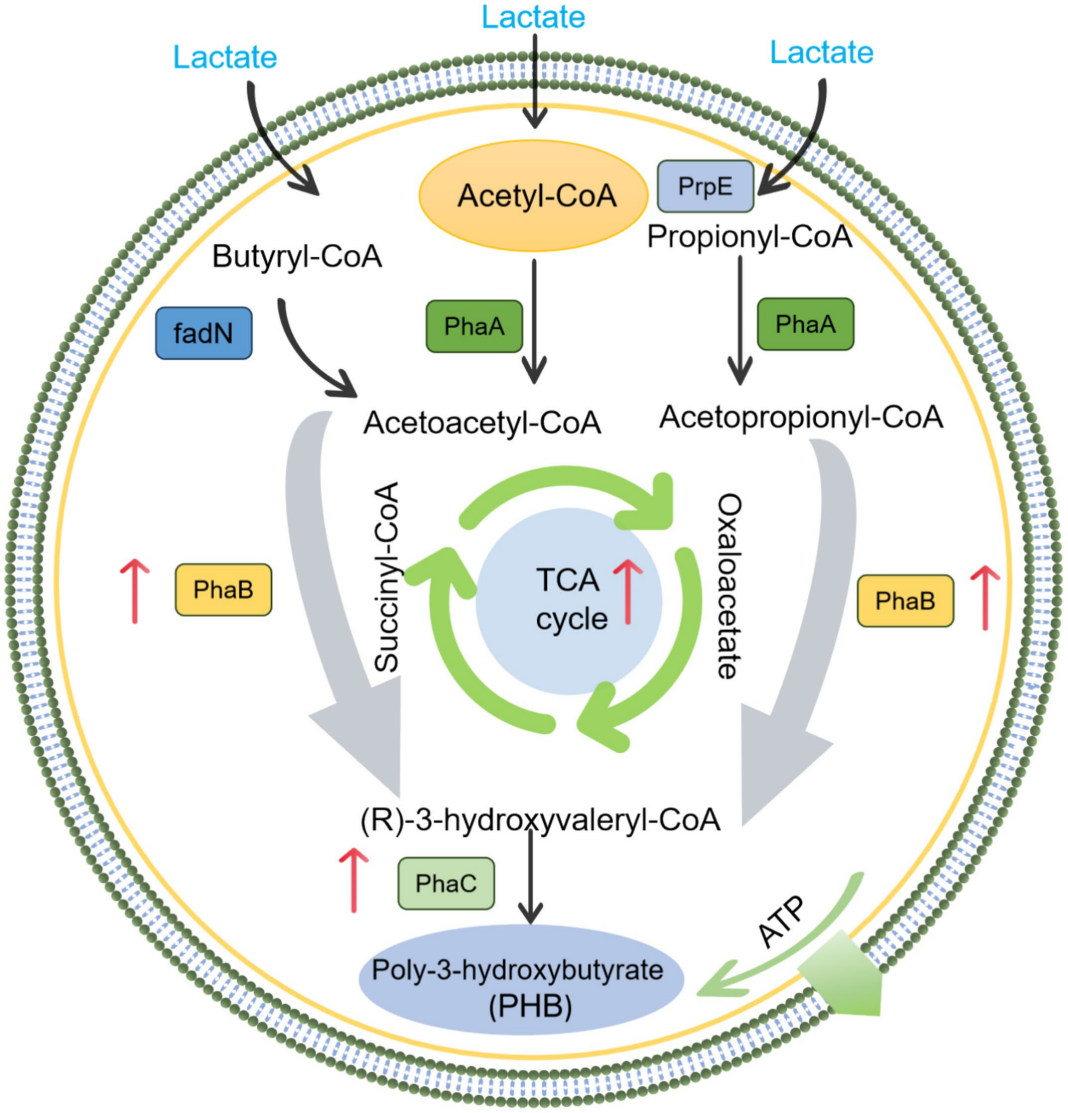
Mechanism of metal ion enhanced PHB accumulation by PSB.

Based on published literatures, the expression of some key genes related to PHB production were selected to further explore the enhancement mechanism of metal ions (Zhang et al., 2025; Wang et al., 2025). As shown in Figure 7, the appropriate concentration of metal ions significantly promoted PhaC, acetoacetyl-CoA reductase, 3-hydroxyacyl-CoA dehydrogenase, butyryl-CoA dehydrogenase, pyruvate dehydrogenase, and L-lactate dehydrogenase. While the excessive metal ions significantly inhibited the expression of these functional genes. Among these functional genes, PhaC and acetoacetyl-CoA reductase are key enzymes in the metabolic pathway for PHB production. The abundance of PhaC for appropriate Fe^3+^ and Mn^2+^ were 2.6 and 3.3 times higher than that of excess metal ions, respectively, which also corresponded to PSB biomass and PHB accumulation. Zhang et al. (2025) reported that the expression of PhaC was significantly upregulated by 2.7 times during the improvement of PSB biomass and PHB accumulation by low-intensity ultrasound. Meanwhile, the abundance of acetoacetyl-CoA reductase for appropriate metal ions were higher than that of excess metal ions. In addition, L-lactate dehydrogenase can catalyze the conversion of lactate to pyruvate. The abundance of L-lactate dehydrogenase for appropriate metal ions were higher than that of excess metal ions, which also corresponded to the lactate utilization (Figure 8).

### 3.4 Enhancement mechanisms

Appropriate Fe^3+^ and Mn^2+^ promoted the PSB biomass and PHB accumulation, while excessive iron and manganese ions inhibited them. Fe^3+^ altered the permeability of cell membranes, making it easier for nutrients and metabolites to pass through the cell membrane, potentially enhancing the metabolic activity of microorganisms (Worms et al., 2006; Wang, 2024). Fe^3+^ also promoted the capture and conversion of photosynthetic energy, drive the synthesis of PHB precursors, and directly activated key enzyme systems involved in PHB synthesis (Karimirad et al., 2024). Fe^3+^ is important coenzyme factors for catalase, which may enhance the antioxidant defense system of PSB, reduce reactive oxygen species (ROS), and prevent PHB depolymerase activation (Wu et al., 2012). Meanwhile, Mn^2+^ could ensure the electron transfer chain flux, provide sustained reducing power (NADPH) for PHB synthesis, and selectively regulated the flow of carbon metabolism toward PHB synthesis (Yang et al., 2021). As cofactor for pyruvate carboxylase, Mn^2+^ can replenish oxaloacetate for succinate synthesis, and can maintain redox homeostasis critical for PHB synthase activity (Lin et al., 2025). In this study, the appropriate concentration of Fe^3+^ and Mn^2+^ significantly promoted the relative abundance of *Rhodopseudomonas*, corresponding to higher PSB biomass production. Meanwhile, the appropriate concentration of Fe^3+^ and Mn^2+^ enhanced the metabolic characteristics of PSB, such as carbon metabolism, carbon fixation, photosynthesis, pyruvate metabolism, etc., further enhancing the PSB metabolic activity and PHB accumulation. In addition, the appropriate concentration of Fe^3+^ and Mn^2+^ upregulated the expression of key functional genes (PhaC and acetoacetyl CoA reductase), which further corresponded to the accumulation of PHB.

## 4 Conclusion

This study aims to investigate the enhancement effect of Fe^3+^ and Mn^2+^ on the PHB production from kitchen waste digestate with PSB, and to reveal their enhancement mechanism. The main conclusions are as follows:

(1) The optimal concentrations of Fe^3+^ and Mn^2+^ enhanced PSB biomass and PHB accumulation. Biomass of PSB reached 2366.3 and 2109.2 mg/L, respectively under the optimal Fe^3+^ and Mn^2+^ concentrations, and PHB content reached 46.0 and 43.8%, respectively.
(2) PSB showed good removal for lactate, SCOD, and ammonia nitrogen in the kitchen waste digestate, exceeding 90, 90, and 70% under optimal conditions for Fe^3+^ and Mn^2+^ concentrations.
(3) Addition of Fe^3+^ and Mn^2+^ reduced the diversity of PSB microbial communities and altered their composition. *Rhodopseudomonas* dominated the microbial community in the photofermentation system and was the main contributor to SCOD removal and PHB accumulation.
(4) Fe^3+^ and Mn^2+^ optimized the metabolic pathways of PSB, such as carbon metabolism, carbon fixation, photosynthesis, pyruvate metabolism, etc. The expression upregulation of PhaC and acetoacetyl CoA reductase enhanced the synthesis of PHB.
(5) The mechanism of metal ion enhanced PHB production by PSB was to increase the relative abundance of key microorganisms, improve metabolic functions, and promote the expression of key functional genes.

However, the mechanism of enhancement in this study was only based on 16S rRNA sequencing, and the enhancement mechanism of Fe^3+^ and Mn^2+^ in PHB production by PSB still needs to be further revealed. Combined with multi-omics techniques will be conducive to reveal the enhancement mechanism in depth. Meanwhile, metal ion-enhanced PHB production with PSB using kitchen waste digestate is still at the lab level, and the scale-up experiments need to be further carried out. In addition, the monomer composition of PHB should be explored, which will be more conducive to reveal the mechanism.

## Data Availability

The original contributions presented in the study are publicly available. This data can be found in here: https://www.ncbi.nlm.nih.gov/, accession number PRJNA1295749.
