# Supplementary material for: Metal ion-enhanced photosynthetic bacteria for bioplastic production from kitchen waste digestate: performance and mechanism
Source: Front Microbiol. 2025 Aug 12;16:1645573. doi: 10.3389/fmicb.2025.1645573 (PMC12378262; doi:10.3389/fmicb.2025.1645573)
Supplement: Supplementary file 1 [file Supplementary_file_1.docx]

**Metal ion-enhanced photosynthetic bacteria for bioplastic production from kitchen waste digestate: Performance and mechanism**

**Wei Zhao^a^, Huize Liu^b^, Jingyi Li^b^, Shiqi Liu^b^, Xue Tao^c^,** **Li Sun^b^, Longyi Lv^b^, Jinsong Liang^b,^ *, Guangming Zhang^b,^ ***

*^a^* School of Heilongjiang River and Lake Management, Heilongjiang University, Harbin, 150080, China

*^b^* School of Energy & Environmental Engineering, Hebei University of Technology, Tianjin, 300130, China

*^c^* Department of Resources and Environment, Moutai Institute, Luban Avenue, Renhuai City, Guizhou Province 564507, China

^*^**Correspondence:**

J. S. Liang, G.M. Zhang.

E-mail address: 2023922@hebut.edu.cn (J. S. Liang), [2020017@hebut.edu.cn](mailto:2020017@hebut.edu.cn) (G.M. Zhang).

**Text S1 Microbial analysis**

The E.Z-N.A ® DNA kit (Omega Bio tek, Norcross, GA, U.S.) was used to extract the DNA of samples. 338F and 806R were used as primers for polymerase chain reaction amplification of the variable region of V3-V4. Polymerase chain reaction products were used for 2 % agarose gel electrophoresis to analyze the amplification of the target band, and were purified with the AxyPrepDNA gel recovery kit (AXYGEN, USA). The products of polymerase chain reaction obtained above were sequenced on the Illumina Miseq PE300 platform (Illumina, San Diego, U.S.) at the paired-end to construct a microbial diversity sequencing library. Each sample obtained was classified according to phylum and genus, and the sequencing data were analyzed on the online free platform Majorbio Cloud platform (www.majorbio.com). The clusters of orthologous groups and kyoto encyclopedia of genes and genomes orthology (KO) information obtained for each operational taxonomic units were then used to calculate the respective abundance. PICRUSt 2 was used to obtain the relative abundance of functional genes related to PHB production.

Table S1 Indices of kitchen waste before and after natural anaerobic fermentation

| Indices | Before fermentation | After fermentation |
| --- | --- | --- |
| pH | 7.3 | 3.8 |
| TS (%) | 16.3 | 14.1 ± 0.07 |
| VS (%) | 13.5 | 11.1 ± 0.2 |
| SCOD (mg/L) | 22988 ± 689.6 | 56443.3 ± 547.9 |
| TP (mg/L)  NH_4_^+^-N (mg/L)  Protein (mg/L)  Polysaccharide (mg/L) | 10.1 ± 0.7  21.1 ± 3.3  3176.1 ± 191.9  7685.9 ± 140.9 | 49.9 ± 1.2  190.2 ± 5.6  4716.7 ± 146.3  8820.9 ± 426.9 |
| Lactate (mg/L) | 126.1 ± 6.4 | 11634.6 ± 426.9 |


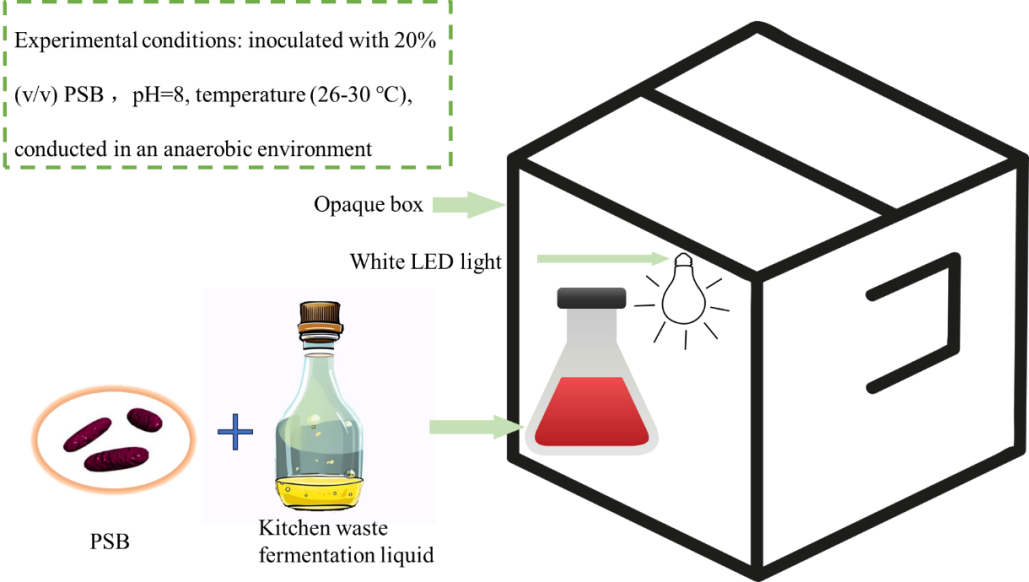


Fig. S1 Experimental setup diagram


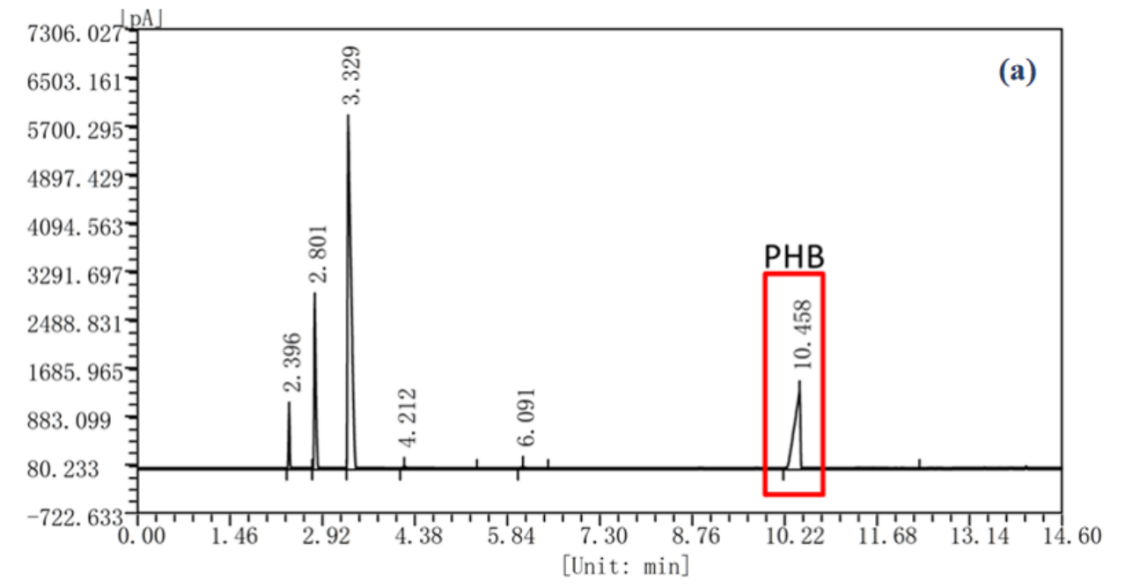


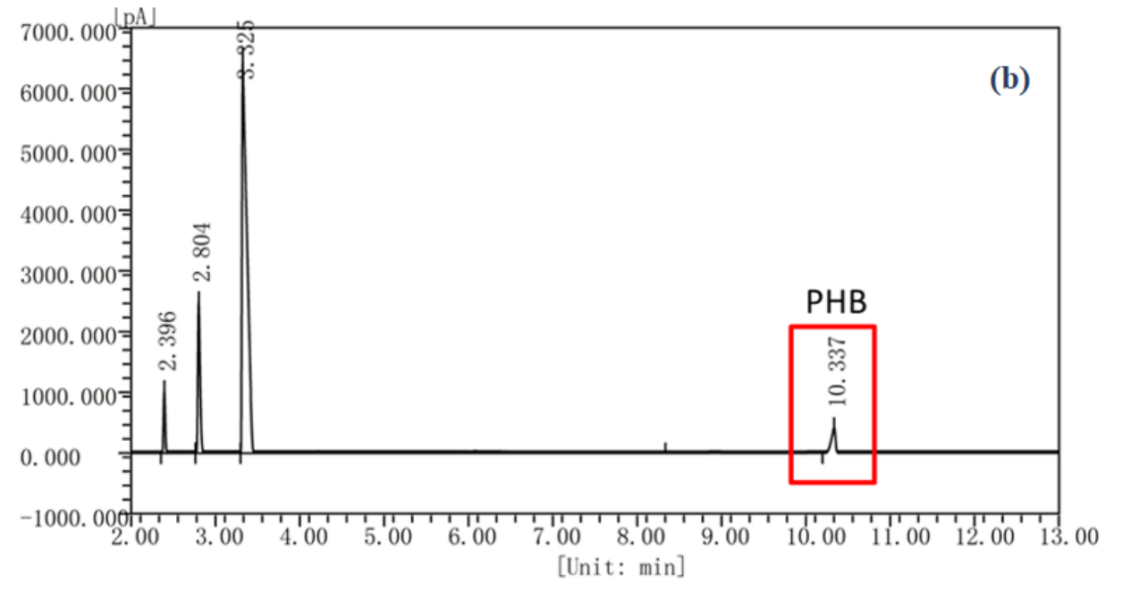


Fig. S2 Gas chromatogram of PHB standard (a) and representative PSB sample (b)
